# Nuclear Receptor NR4A2 Orchestrates Th17 Cell-Mediated Autoimmune Inflammation via IL-21 Signalling

**DOI:** 10.1371/journal.pone.0056595

**Published:** 2013-02-21

**Authors:** Ben J. E. Raveney, Shinji Oki, Takashi Yamamura

**Affiliations:** Department of Immunology, National Institute of Neuroscience, National Center of Neurology and Psychiatry, Kodaira, Tokyo, Japan; Hannover Medical School, Germany

## Abstract

IL-17-producing CD4^+^ T helper 17 (Th17) cells are pathogenic in a range of human autoimmune diseases and corresponding animal models. We now demonstrate that such T cells infiltrating the target organ during the induction of experimental autoimmune encephalomyelitis (EAE) and experimental autoimmune uveoretinitis (EAU) specifically express NR4A2. Further, we reveal a critical involvement of NR4A2 in Th17 cell functions and Th17 cell-driven autoimmune diseases. When NR4A2 expression was blocked with siRNA, full Th17 differentiation was prevented *in vitro*: although cells expressed the master Th17 regulator, RORγt, they expressed reduced levels of IL-23R and were unable to produce IL-17 and IL-21. Notably, Th17 differentiation in the absence of NR4A2 was restored by exogenous IL-21, indicating that NR4A2 controls full maturation of Th17 cells via autocrine IL-21 signalling. Preventing NR4A2 expression *in vivo* by systemic treatment with NR4A2-specific siRNA also reduced Th17 effector responses and furthermore protected mice from EAE induction. In addition, the lack of disease was associated with a reduction in autocrine IL-21 production and IL-23R expression. Similar modulation of NR4A2 expression was also effective as an intervention, reversing established autoimmune responses and ameliorating clinical disease symptoms. Thus, NR4A2 appears to control Th17 differentiation and so plays an essential role in the development of Th17-mediated autoimmune disease. As NR4A2 is also upregulated during human autoimmune disease, targeting NR4A2 may provide a new therapeutic approach in treating autoimmune disease.

## Introduction

T helper (Th) cells responding to self-antigens generate pathogenic inflammatory responses in target organs, leading to local damage and so generate organ-specific autoimmune diseases. It was previously thought that CD4^+^ interferon (IFN)-γ-secreting Th1 cells were critical in inducing autoimmune damage to the central nervous system (CNS) in human multiple sclerosis (MS) and its animal model, experimental autoimmune encephalomyelitis (EAE) [1]. However, the discovery of pathogenic IL-17-secreting Th17 cells as a separate cell lineage opened the door to new research directions towards understanding the development of autoimmune inflammation [2]–[4]. It is now understood that both Th1 and Th17 cells mediate autoimmune responses in rodents [5]–[8] as well as in humans [9], [10]. Regarding the development of EAE, it has recently been proposed that the major proportion of T cells producing inflammatory cytokines, including IFN-γ, may in fact be T cells that had previously produced IL-17 [11]. Thus, manipulation of Th17 cells might prove effective in controlling complex autoimmune disease processes involving both Th1 and Th17 cells.

Naïve CD4^+^ T cells differentiate into Th1 cells under the influence of IL-12, whereas TGF-β in combination with IL-6 is appreciated as the classical Th17-differentiating cytokine milieu [12], [13]. Recently, however, Th17 differentiation pathways that do not depend on IL-6 or TGF-β have also been described [14]–[16]. In contrast, *in vivo* studies demonstrate that IL-23 plays a critical role in promoting generation of Th17 cells. Indeed, Th17-mediated autoimmune disease is greatly reduced or prevented in the absence of IL-23 signalling [17], [18].

NR4A2, also known as Nurr1, is an orphan nuclear receptor [19]–[22], and its function in dopaminergic neuron signalling has been widely known. Increasing evidence suggests the role of NR4A2 in inflammatory responses during arthritis and psoriasis [23], [24], and NR4A2 may also serve as a regulatory element for reducing immune-mediated tissue damage [25]. We have previously reported that NR4A2 is among the genes expressed by circulating T cells that are highly upregulated in patients with multiple sclerosis (MS) and that NR4A2 is also induced in T cells during rodent EAE [26], [27]. We also demonstrated that forced NR4A2 expression enhanced non-specific production of Th1 and Th17 cytokines although further confirmation was needed to confirm the role of NR4A2 in T cell functions.

In this study, we firstly show that NR4A2 is strikingly upregulated by IL-17-secreting Th17 cells infiltrating the target organ of EAE and experimental autoimmune uveoretinitis (EAU), the murine model of posterior uveitis. Using siRNA knockdown techniques, we demonstrate that NR4A2 is dispensable for induction of the Th17 cell transcription factor RORγt in T cells, but is critically required for the *in vitro* generation of fully functional Th17 cells capable of producing IL-17 and IL-21, and expressing the IL-23 receptor (IL-23R). Notably, addition of exogenous IL-21 was able to circumvent the requirement for NR4A2 in Th17 differentiation, and restore the expression of IL-23R and IL-17. Furthermore, NR4A2 knockdown *in vivo* by injection of NR4A2 siRNA either before or after the onset of CNS infiltration ameliorated EAE. Taken together, these data suggest that T cell NR4A2 expression is a hallmark of Th17 cell-mediated pathology and show for the first time that systemic injection of a NR4A2-targeting drug may be a treatment option for Th17-cell mediated diseases.

## Materials and Methods

### Animals and EAE/EAU Induction

Female C57BL/6J mice (CLEA Laboratory Animal Corp., Tokyo, Japan) aged 8–10 weeks were maintained in specific pathogen–free conditions in accordance with institutional guidelines. This study and all protocols used were approval by the Committee for Small Animal Research and Animal Welfare (National Center of Neurology and Psychiatry). Procedures were carried out under institutional guidelines and all efforts were made to minimize animal suffering. For EAE induction, mice were injected subcutaneously with 100 µg MOG_35–55_ peptide (synthesized by Toray Research Center, Tokyo, Japan) and 1 mg heat-killed mycobacterium tuberculosis H37RA emulsified in complete Freund’s adjuvant (Difco, KS, USA). 200 ng Pertussis toxin (List Biological Laboratories, USA) was injected intraperitoneally (*i.p.*) on days 0 and 2 after immunization. EAE was clinically scored daily (0, no clinical signs; 1, partial tail paralysis; 2, flaccid tail; 3, partial hind limb paralysis; 4, total hind limb paralysis; 5, hind and fore leg paralysis) [28]. EAU was induced as previously described by immunization with 500 µg human interphotoreceptor binding protein (IRBP)_1–20_ peptide (synthesized by Toray Research Center) in CFA per mouse plus 1 µg Pertussis toxin [29]. EAU disease severity was monitored by enumeration of the retinal-infiltrating cell number as previously described [30]. Diabetes was induced as previously described [31] by 5 daily *i.p.* doses of 40 mg/kg streptozotocin (STZ; Sigma, Tokyo, Japan) and the urine glucose level was determined daily with Diastix (Bayer, Tokyo, Japan).

### siRNA Treatment

NR4A2-specifc siRNA (sense – GGACAGCAGUCCUCCAUUAAUU, anti-sense - UUAAUGGAGGACUGCUGUCCUU) and matching scrambled control sequence siRNA were synthesized by Takara (Shiga, Japan) or Koken (Tokyo, Japan). siRNA was transfected into cells using a mouse CD4 nucleofector kit with an Amaxa electroporator (Lonza, Basel, Switzerland) according to the manufacturer’s instructions. For systemic *in vivo* administration, siRNA was stabilized in atelocollagen using an AteloGene kit according to the manufacturer’s instructions (Koken) and 10 µg siRNA per mouse was injected intravenously.

### Cell Isolation and Purification

Single cell splenocyte and lymph node cell suspensions were generated by mechanical disruption of tissues. CNS-infiltrating lymphocytes were isolated from spinal cords and brains as previously described [28]. Briefly, tissue was cut into small pieces and digested for 40 minutes at 37°C in RPMI 1640 media (Invitrogen, Tokyo, Japan) supplemented with 1.4 mg/ml Collagenase H and 100 µg/ml DNase I (Roche, Tokyo, Japan). Resulting tissue homogenates were forced through a 70 µm cell strainer and leukocytes were enriched using a discontinuous 37%/70% percoll density gradient centrifugation (GE Healthcare Life Sciences, Tokyo, Japan). Retinal-infiltrating cells and pancreatic-infiltrating cells were isolated by enzymatic digestion as previously described [32], [33].

T cells were purified using a CD4 T cell MACS isolation kit with an AutoMACS separator according to the manufacturer’s instructions (Miltenyi Biotech, Bergisch Gladbach, Germany). Where required, naïve CD4^+^CD44^−^CD25^−^CD62L^high^ T cells or memory CD4^+^CD44^+^CD25^−^CD62L^low^ T cells were further sorted using a FACS ARIA (BD Cytometry Systems, NJ, USA). For sorting of live cytokine-secreting cells, cytokine secretion assay kits (Miltenyi Biotech) were used according to the manufacturer’s instructions. Briefly, cells were restimulated with 5 ng/ml PMA +500 ng/ml ionomycin (both Sigma-Aldrich, Tokyo, Japan) and anti-IL-17 and anti-IFN-γ capture antibodies were added for the final 45 minutes of culture. Secondary fluorochrome-conjugated antibodies were added to visualize captured cytokines and cells were sorted using a FACS ARIA flow cytometer.

### Cell Culture

Culture media was DMEM supplemented with 10% FCS, 2 mM L-glutamine, 100 U/ml penicillin-streptomycin, and 50 µM 2-Mercaptoethanol (all Invitrogen). Where indicated, cells were activated with 2 µg/ml immobilized CD3-specific mAb (2C-11) and 1 µg/ml CD28-specific mAb (BD Pharmingen, Tokyo, Japan). Polarizing conditions were as follows: Th1, +10 ng/ml IL-12 (PeproTech, London, UK) and 10 µg/ml IL-4-specific mAb (HB188); Th17, 3 ng/ml TGF-β (R & D Systems, Minneapolis, USA), 20 ng/ml IL-6 (PeproTech), 20 ng/ml IL-23 (R & D Systems), 10 µg/ml IFN-γ-specific mAb, and 10 µg/ml IL-4-specific mAb.

### Assessment of Cell Function

Cytokine concentrations in supernatants were measured by ELISA as follows: IL-17 using a mouse IL-17 DuoSet (R&D Systems), IL-21 using an IL-21 MaxLegend kit (Biolegend, San Diego, USA), and IFN-γ using a mouse IFN-γ ELISA antibody pair (BD Biosciences). Other cytokines were assessed using a FlowCytomix cytometric bead array (Bender MedSystems, Vienna, Austria) according to the manufacturer’s instructions. Proliferation was determined by incubation with [^3^H]-thymidine (1 µCi/well) for the final 12 hours of culture and incorporation of radioactivity was assessed with a β-1205 counter (Pharmacia Biotech, Freiburg, Germany). For intracellular staining, cells were restimulated with 5 ng/ml PMA +500 ng/ml ionomycin (both Sigma-Aldrich) in the presence of Golgi Stop (BD Biosciences) for 5 hours, before surface staining and fixing/intracellular staining using a Foxp3 staining kit (eBioscience, San Diego, CA, USA) according to the manufacturer’s instructions. Antibodies were sourced from BioLegend (San Diego, USA), except for anti-cytoplasmic IL-23R (Millipore, Tokyo, Japan).

### RNA Extraction and Quantitative RT-PCR

Total RNA was extracted from cell populations using an RNeasy Mini Kit or FastLane kit (Qiagen, Maryland, USA) according to the manufacturer’s instructions. cDNA was prepared using a first-strand cDNA Kit (Takara). Quantitative real time PCR was performed with a Light Cycler-FastStart DNA Master SYBR Green I kit using a LightCycler instrument (Roche Diagnostics, Tokyo, Japan) or with a Power SYBR green master mix using an ABI 7300 real time PCR instrument (Applied Biosystems, Warrington, UK). Primers used were as follows: GAPDH, forward AACGACCCCTTCATTGAC, reverse TCCACATACTCAGCAC; RORγt, forward TGTCCTGGGCTACCCTACTG, reverse GTGCAGGAGTAGGCCACATT; t-bet, forward GCCAGGGAACCGCTTATATG, reverse GACGATCATCTGGGTCACATTGT; IL-21, forward TCATCATTGACCTCGTGGCCC, reverse ATCGTACTTCCACTTGCAATCCC; IL-23R, forward TCAGTGCTACAATCTTCAGAGGACAT, reverse GATGGCCAAGAAGACCATTCC; IL-17A, forward ATCCCTCAAAGCTCAGCGTGTC, reverse GGGTCTTCATTGCGGTGGAGAG; and Foxp3, forward TTCTCACAACAAGGCCACTTG, reverse CCCAGGAAAGACAGCAACCCT. Gene expression values were normalized to the expression of the GAPDH housekeeping gene.

### Statistical Analyses

Statistical significance of differences was tested using a Mann Whitney U test unless otherwise stated. p<0.05 was considered significant.

## Results

### Organ-specific Autoimmune Diseases EAE and EAU Accompany NR4A2 Regulation in T cells

**Figure 1:**
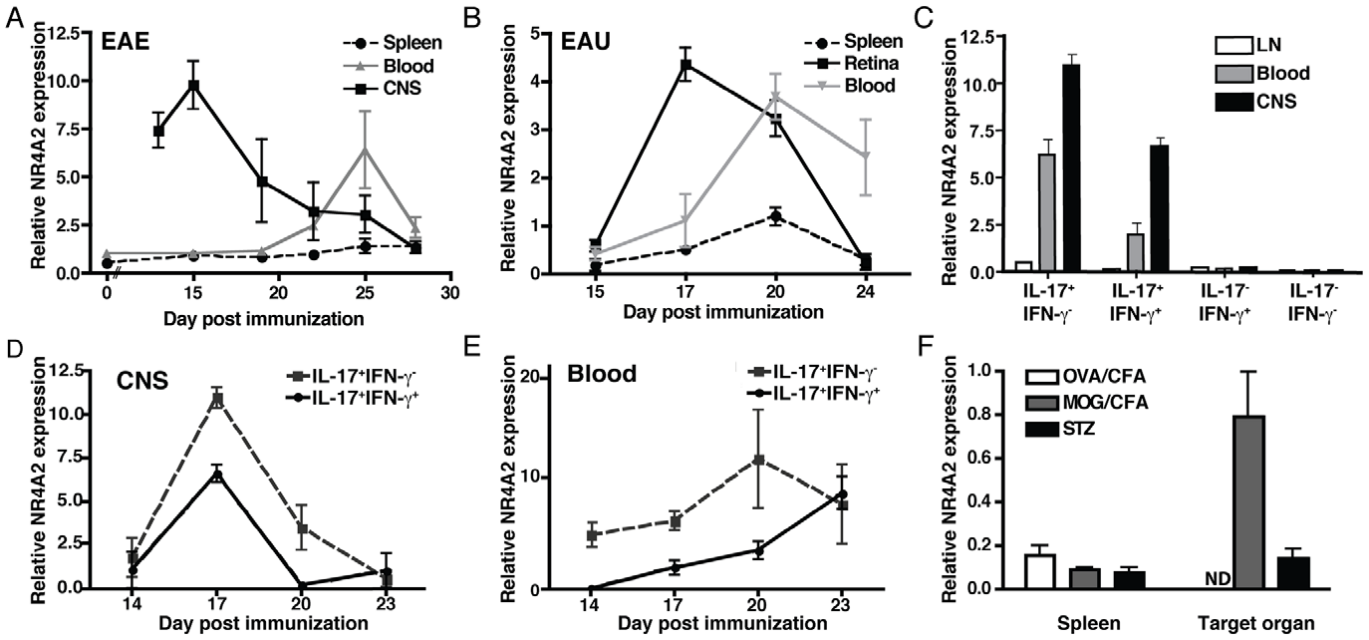
Autoimmune induction of NR4A2 in CD4^+^ T cells is associated with IL-17-secreting T cells. EAE or EAU was induced in C57BL/6 mice by immunization with MOG_35–55_ or IRBP_1–20_ peptide in CFA, respectively. CD4^+^ T cells were purified from spleen, blood, or target organ (CNS or retina) on the indicated days and RNA was isolated. **A** and **B:** NR4A2 expression was quantified by real time PCR relative to GAPDH for T cells from EAE (A) or EAU (B). Timepoints correspond to a minimum of 5 animals and data are representative of 3 independent experiments. CD4^+^ T cells from mice with EAE were restimulated with PMA/ionomycin for 3 hours and 4 populations of cytokine secreting cells (IL-17+IFN-γ-, IL-17+IFN-γ+, IL-17-IFN-γ+, and IL-17-IFN-γ-) were sorted by flow cytometry using IFN-γ and IL-17 cytokine secretion assay kits. **C:** NR4A2 expression by populations of cytokine-secreting CD4^+^ T cells was quantified by real time PCR at day 15 post-EAE induction for lymph nodes (LN) and CNS-infiltrating cells (CNS), and day 25 for blood T cells. **D** and **E:** NR4A2 expression by IL-17^+^IFN-γ^−^ or IL-17^+^IFN-γ^+^ CNS-infiltrating T cells (**D**) or blood T cells (**E**) was measured by real time PCR at a range of timepoints. Data are representative of 2 independent experiments. **F:** Th1-mediated diabetes was induced in C57BL/6 mice by 5 daily low dose STZ treatments. Other groups of C57BL/6 mice were immunized with peptides in CFA plus PTX either OVA_323–339_ (OVA/CFA) or MOG_35–55_ (MOG/CFA). On day 22, NR4A2 expression was assessed by real time PCR amongst CD4^+^ T cells from spleen and leukocytes isolated from the relevant target organ (ND, OVA/CFA; CNS, EAE; pancreas, STZ). Timepoints correspond to a minimum of 5 animals and data are representative of 2 independent experiments.

We previously reported T cell expression of NR4A2 during EAE [27]. Here we tested whether or not NR4A2 overexpression is common to T cells involved in autoimmune diseases. We detected NR4A2 upregulation amongst T cells during the development of EAE and EAU, a Th1/Th17-mediated autoimmune disease of the retina: T cell expression of NR4A2 was observed from the earliest stages of both EAE and EAU in CD4^+^ T cell infiltrates in the target organ and, as well as a later NR4A2 upregulation amongst circulating blood CD4^+^ T cells (Fig. 1A&B) after the peak of clinical disease (Fig. S1A&B). Although T cells from secondary lymphoid tissue have the potential to induce EAE or EAU when they are adoptively transferred after being stimulated *in vitro* [34], no significant NR4A2 expression was detected amongst lymph node or splenic T cells at any time examined.

### NR4A2 Expression is Associated with IL-17 Producing T cells in EAE

We previously observed that forced expression of NR4A2 in T cells could enhance production of both IL-17 and IFN-γ [27]. However, since this observation was made under non-physiological conditions, we attempted to verify if this finding had physiological meaning *in vivo*. Using a cytokine secretion assay, we separated populations of live CD4^+^ T cells from the blood and CNS of EAE mice based on their production of IFN-γ and IL-17 and measured the expression levels of NR4A2 in each population. Strikingly, in the early phase of EAE, NR4A2 transcripts were detected in those T cells that produced IL-17, either alone or in combination with IFN-γ (Fig. 1C), but not in those that produced IFN-γ alone or neither IFN-γ nor IL-17. Similar findings were observed in T cells isolated from the retina during the early stages of EAU. NR4A2 expression by IL-17-secreting T cells in EAE was detected first in the target organ then later in the blood (Fig. 1D&E), which was concordant with the kinetics of NR4A2 expression in total lymphocytes (Fig. 1A).

### NR4A2 Expression is not Detected in STZ-induced Diabetes or following OVA Immunization

We next measured NR4A2 expression in T cells from streptozotocin (STZ)-induced autoimmune diabetes [35], in which autoimmune Th1 cells but not Th17 cells are thought to play a pathogenic role [31]. Repeated administration of low-dose streptozotocin (STZ) induced anti-pancreatic autoimmunity accompanied with clinical diabetes by day 10 (Fig. S1C). Consistent with a previous report [35], splenocytes as well as pancreata-infiltrating T cells produced raised levels of IFN-γ, but not IL-17, after *in vitro* stimulation (Fig. S1D). Unlike CNS-infiltrating T cells in EAE, NR4A2 upregulation was not detected amongst pancreata-infiltrating T cells (Fig. 1F). We also examined blood T cells from these mice and detected no NR4A2 upregulation at any time (Fig. S1E). Furthermore, we examined if NR4A2 upregulation might be induced by active immunization with any antigen in CFA. However, immunization with OVA in CFA, using the same protocol for inducing EAE with MOG peptide, did not lead to NR4A2 upregulation.

### NR4A2 Expression is Required for IL-17 Production by RORγt^+^ T cells

**Figure 2:**
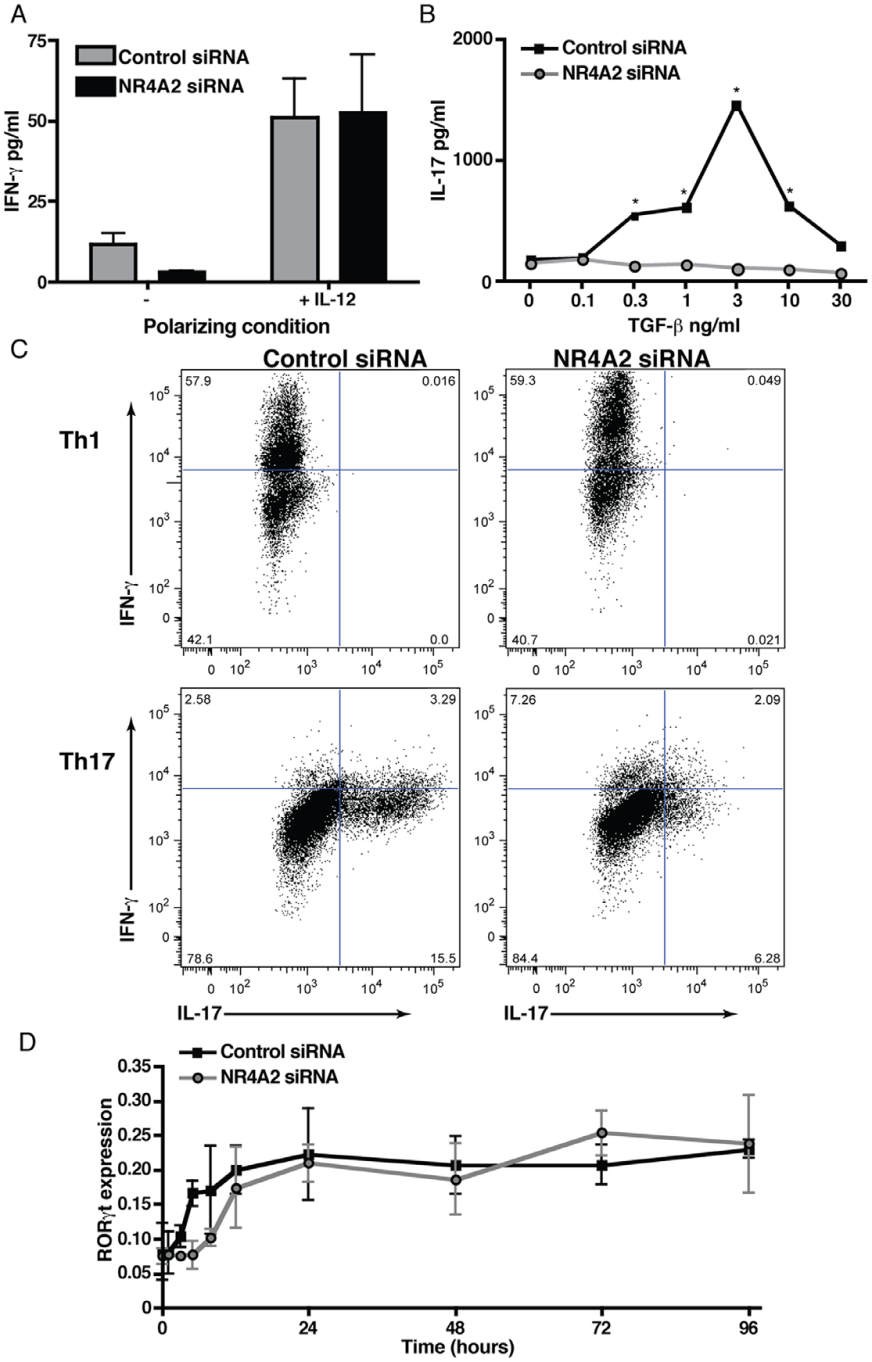
NR4A2 knockdown prevents IL-17 secretion but not RORγt upregulation. Naïve CD4^+^ T cells were transfected by electroporation with NR4A2-specific siRNA or scrambled control siRNA. Cells were then activated with 5 µg/ml plate-bound CD3-specific mAb and 0.5 µg/ml soluble CD28-specific mAb. **A:** IFN-γ production by cells activated in the presence or absence of 10 ng/ml IL-12 after 96 hours of culture. **B:** IL-17 production by cells activated in the presence of 20 ng/ml IL-6, 20 ng/ml IL-23, and TGF-β at a range of concentrations after 96 hours of culture. Significant differences between control and NR4A2 siRNA-treatments were tested with a student’s t-test, *p<0.05. **C:** IL-17 and IFN-γ intracellular cytokine staining for transfected T cells (control siRNA, left plots; NR4A2 siRNA, right plots) cultured for 96 hours in the presence of 10 ng/ml IL-12 (Th1 conditions, top row plots) or 20 ng/ml IL-6, 20 ng/ml IL-23, and 3 ng/ml TGF-β (Th17 conditions, bottom row plots). **D:** RORγt RNA expression as measured by real time PCR by activated T cells cultured under Th17 polarizing conditions at a range of timepoints. Data are representative of 5 independent experiments.

As NR4A2 expression appeared to be associated with IL-17-secreting pathogenic T cells, we speculated that NR4A2 might function in the process of Th17 cell differentiation. Using NR4A2-specific siRNA, we investigated *in vitro* if CD4^+^ T cells differentiate normally into Th1 or Th17 cells in the absence of NR4A2 regulation. Activation of T cells leads to a rapid and transient upregulation of NR4A2 that could be prevented by transfection with NR4A2 siRNA (Fig. S2A). When NR4A2 expression was silenced in this manner, naïve CD4^+^ T cells were able to differentiate into IFN-γ-producing cells (Fig. 2A&C), excluding a requirement for NR4A2 in Th1 cell development. However, blocking NR4A2 upregulation with siRNA greatly reduced Th17 differentiation driven by any concentrations of TGF-β, as assessed by an absence of IL-17 production (Fig. 2B&C), instead there was an increase in IFN-γ-secreting T cells. We also noted that NR4A2 knockdown did not significantly reduce the proliferation of CD4^+^ T cells under any polarizing conditions tested (Fig. S2B), indicating that NR4A2-specific siRNA is unlikely to prevent IL-17 production by affecting cell survival. Intriguingly, despite the lack of IL-17 production in the absence of NR4A2, T cells did upregulate RORγt, the hallmark transcription factor of Th17 cells, to levels comparable to fully functional Th17 cells (Fig. 2D).

### Lack of IL-17 in the Absence of NR4A2 does not Result from the Action of Foxp3

A previous study has described a failure of RORγt-expressing Th17 cells to secrete IL-17 resulting from a direct inhibitory interaction between RORγt and the transcription factor Foxp3 [36]. To examine if a similar mechanism, involving Foxp3, is applicable to interpreting our results, we measured the level of Foxp3 expression during NR4A2 knockdown. NR4A2 siRNA did not enhance but reduced Foxp3 expression in both Th17 (TGF-β+IL-6) and regulatory T cell (TGF-β alone) differentiating conditions (Fig. S3A). This finding is consistent with a recent report on a role of NR4A2 for inducing Foxp3 in regulatory T cells [37]. Furthermore, we tested if NR4A2 ablation by siRNA treatment was still effective in preventing IL-17 production when Foxp3 expression was blocked. To do this, we used Foxp3-specific siRNA to prevent Foxp3 expression in T cells that also received either control or NR4A2-specific siRNA. Foxp3 knockdown did not restore IL-17 production in the absence of NR4A2 (Fig. S3B). Thus, NR4A2 appears to be required for IL-17 production by Th17 cells, and the underlying mechanism is independent of RORγt and Foxp3.

### NR4A2 Controls the IL-21-initiated Phase of Th17 Differentiation

**Figure 3:**
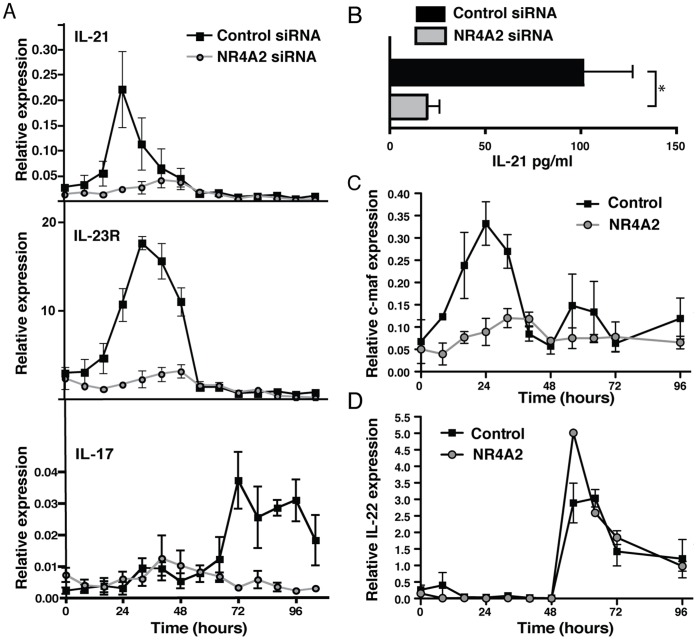
Absence of NR4A2 is associated with a lack of IL-21 production by Th17 cells. Naïve CD4^+^ T cells were transfected by electroporation with NR4A2-specific siRNA or scrambled control siRNA and were activated with 5 µg/ml plate-bound CD3-specific mAb and 0.5 µg/ml soluble CD28-specific mAb in the presence of 20 ng/ml IL-6, 20 ng/ml IL-23, and 3 ng/ml TGF-β. **A:** RNA levels of IL-21, IL-23R, and IL-17 were quantified by real time PCR at the indicated timepoints following activation. Data are representative of 3 independent experiments. **B:** IL-21 supernatant concentration was measured by ELISA at 96 hours. Data are representative of 3 independent experiments. *p<0.05. **C:** RNA expression of c-maf quantified by real time PCR. **D:** RNA expression of IL-22 quantified by real time PCR. Data are representative of 2 independent experiments.

**Figure 4:**
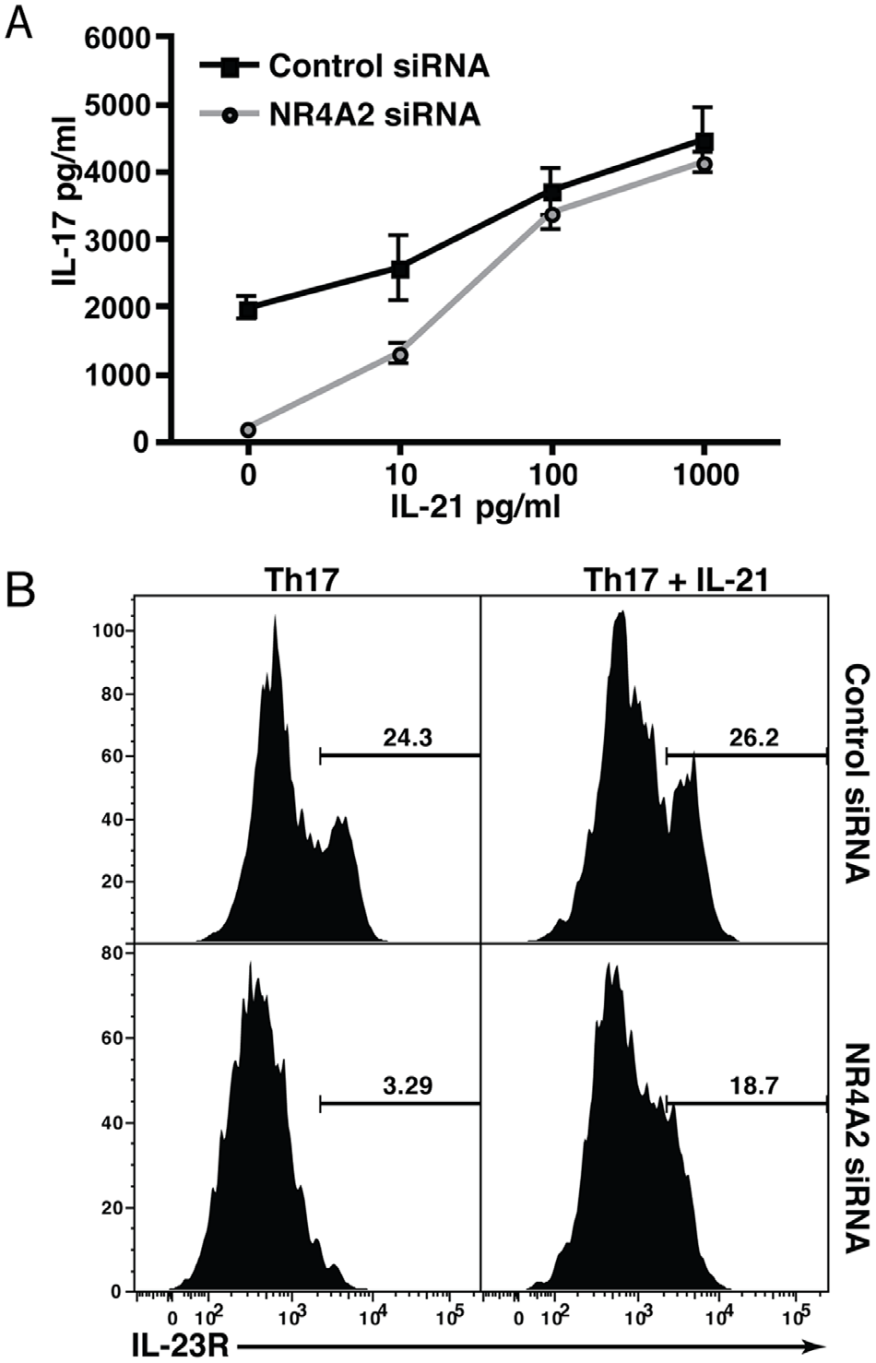
Exogenous IL-21 restores IL-17 production in the absence of NR4A2. Naïve CD4^+^ T cells were transfected by electroporation with NR4A2-specific siRNA or scrambled control siRNA and were activated with 5 µg/ml plate-bound CD3-specific mAb and 0. 5 µg/ml soluble CD28-specific mAb in the presence of 20 ng/ml IL-6, 20 ng/ml IL-23, and 3 ng/ml TGF-β. To some wells, recombinant IL-21 was added as indicated. **A:** IL-17 was measured in the supernatants of control or NR4A2 siRNA-treated T cells by ELISA after 96 hours of culture under Th17 polarizing conditions in the presence or absence of IL-21 at the indicated concentrations. *p<0.05. Data are representative of 3 independent experiments. **B:** IL-23R expression was assessed by intracellular flow cytometric staining after 96 hours of culture under Th17 polarizing conditions in the presence (right plots) or absence (left plots) of 100 pg/ml recombinant IL-21 for control siRNA-treated T cells (top) and NR4A2 siRNA-treated T cells (bottom row). Data are representative of 2 independent experiments.

RORγt expression is required for generation of fully functional Th17 cells. However, when upregulation of NR4A2 is prevented, Th17-polarized RORγt^+^ T cells do not acquire the ability to produce IL-17 (Fig. 2). A possible scenario is that RORγt regulation is an early event in Th17 differentiation [38], whereas later induction of NR4A2 is critical for inducing signals that promote IL-17 production. IL-21-deficient or IL-21R-deficient T cells resemble NR4A2-deficient T cells in that they express RORγt but do not produce IL-17 under Th17 polarizing condition [4], [39]. Thus, we suspected that NR4A2 expression might control IL-17 production via autocrine IL-21 signalling. IL-21 is produced during early Th17 cell differentiation and then acts in an autocrine manner to induce IL-23R upregulation by Th17 cells [38], [40]; the subsequent action of IL-23 produced by myeloid cells then enhances and stabilizes the Th17 cell phenotype via IL-23R. To clarify the kinetics of these key molecules, we evaluated the expression of IL-21, IL-23R, and IL-17 transcripts by RORγt^+^ T cells during *in vitro* Th17 cell differentiation. IL-21 begins to be produced before IL-23R is upregulated, which itself precedes IL-17 expression (Fig. 3A). Interestingly, NR4A2 siRNA transfection strongly inhibited the sequential regulation of IL-21, IL-23R, and IL-17 RNA transcripts (Fig. 3A). We also confirmed the reduction of IL-21 by NR4A2 siRNA treatment at the protein level (Fig. 3B). These data indicate that NR4A2 may be required for IL-21 production and thus in turn control Th17 differentiation. Furthermore, the lack of NR4A2 also blocked c-maf upregulation (Fig. 3C), a transcription factor reported to control IL-21 expression in Th17 development [41]. Finally, Th17 differentiation yields normal IL-22 production in the absence of NR4A2 (Fig. 3D) and the generation of IL-22 has been shown to be related to pathways downstream of RORγt, but independent of c-maf, IL-21, and IL-23 signalling [42]. To test the hypothesis that NR4A2 is required for full Th17 differentiation due to its role in the c-maf/IL-21/IL-23R pathway, we reintroduced this pathway by adding exogenous IL-21 to cultures. Critically, the presence of exogenous IL-21 restored IL-17 secretion by T cells stimulated under Th17 polarizing conditions despite the lack of NR4A2 (Fig. 4A). Additionally, NR4A2-knocked down Th17 cells cultured with IL-21 also expressed equivalent levels of IL-23R to the control Th17 cells (Fig. 4B).

### NR4A2 Controls the Severity of EAE

**Figure 5:**
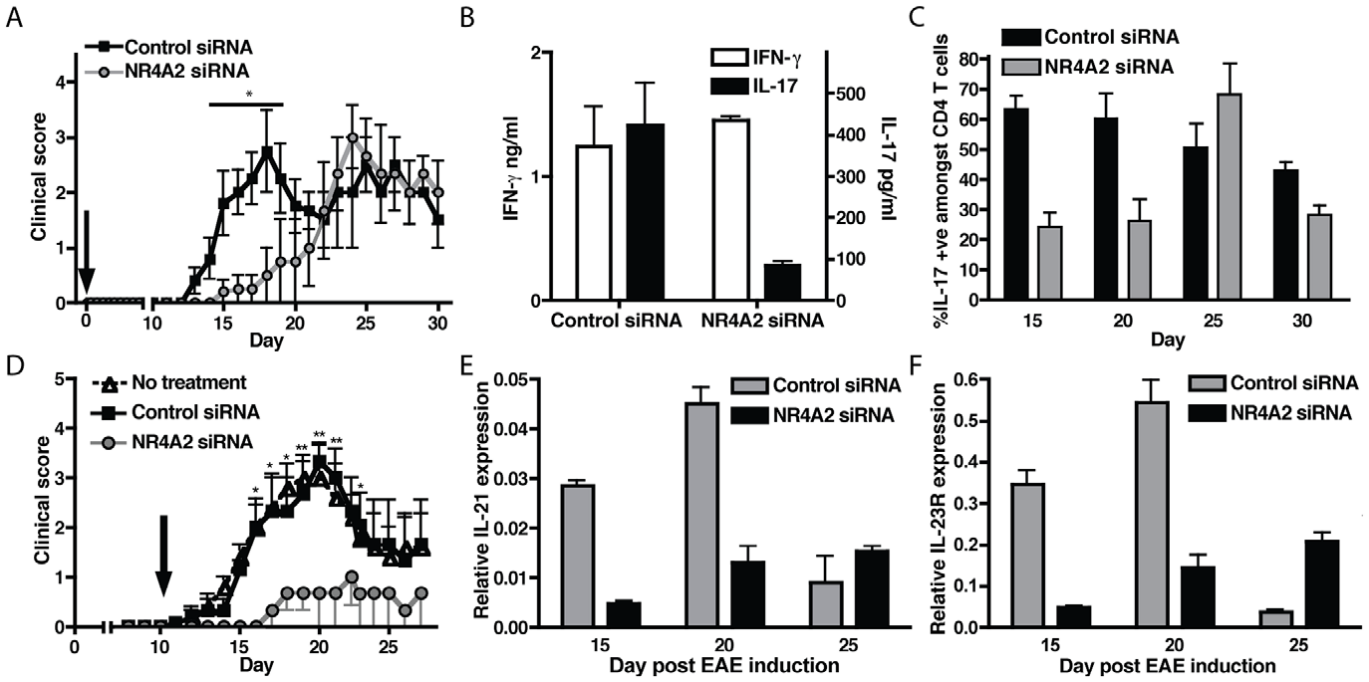
Systemic administration of NR4A2-specific siRNA reduces EAE severity. siRNA, either NR4A2-specific or control, was stabilized in a collagen matrix and administered *i.v.* to groups of C57BL/6 mice at the time of EAE induction. EAE was scored clinically (**A**) and at day 15 post-EAE induction, production of IL-17 and IFN-γ by CNS-infiltrating leukocytes restimulated with 20 µg/ml MOG peptide for 96 hours were assessed by ELISA (**B**). CNS-infiltrating T cells were also assessed for IL-17 production at a range of timepoints by intracellular flow cytometry (**C**). Data are representative of 3 independent experiments. Control or NR4A2-specific siRNA was applied to MOG-immunized mice at day 10 post-disease induction and disease was scored clinically (**D**). Timepoints correspond to a minimum of 5 animals and data are representative of 2 independent experiments. IL-21 and IL-23R expressions amongst CNS-infiltrating T cells were measured by real time PCR (**E&F**). Data are representative of 2 independent experiments. Clinical scores in panels A) and D) were tested with a two-way ANOVA test. *p<0.01, **p<0.001.

Next we tested if NR4A2 also controlled pathogenic Th17 responses in EAE. Administration of NR4A2-specific siRNA on the day of EAE induction was effective at preventing NR4A2 expression by CNS-infiltrating T cells (Fig. S4A). Such systemic blockade of NR4A2 suppressed the onset of clinical EAE (Fig. 5A), accompanied by a reduced ability of CNS-infiltrating CD4^+^ T cells to secrete IL-17 but not IFN-γ (Fig. 5B) when restimulated with the immunizing peptide. NR4A2 siRNA treatment also led to a lower proportion of T cells in the target organ that produced IL-17 upon non-specific restimulation during early timepoints (summarized in Fig. 5C; representative data, Fig. S4B). However, we observed that the effect of NR4A2 siRNA is not persistent, and the mice showed signs of late onset EAE after day 21 (Fig. 5A) accompanied by an increase in the proportion of IL-17^+^ T cells in the CNS (Fig. 5C). Since collagen-stabilized siRNA maintains its suppressive activity *in vivo* for approximately three weeks [43], the later onset of EAE may reflect the reduced potency of the siRNA. We then tested the effect of injection of NR4A2 siRNA at a later timepoint. Interestingly, when the NR4A2 siRNA was given on day 10 post-EAE induction, clinical EAE was greatly reduced, and unlike treatment at day 0, no increase in disease was observed after day 20 (Fig. 5D). These results indicate that NR4A2 targeting siRNA is not only preventative, but also therapeutic against the development of EAE. Furthermore, the CNS-infiltrating T cells also showed reduced expression of IL-21 and IL-23R (Fig. 5E&F), reminiscent of the *in vitro* blocking of NR4A2 during Th17 cell differentiation. Based on these data, we suggest that NR4A2 is a key factor for Th17 differentiation *in vivo* during the initiation of autoimmune responses via its control of IL-21 and IL-23R expression.

## Discussion

In this study, we demonstrate that the orphan nuclear receptor NR4A2 is highly expressed by IL-17-secreting T cells infiltrating the target organ of EAE and EAU. When upregulation of NR4A2 was prevented *in vitro,* Th17-polarizing T cells expressing RORγt did not further differentiate into mature Th17 cell capable of producing IL-21 and IL-17. This inhibition of Th17 cell differentiation was associated with disruption of autocrine IL-21 signalling. *In vitro* analysis showed that adding exogenous IL-21 restored the ability of the NR4A2 knocked-down Th17 cells to express IL-23R and produce IL-17. Furthermore, *in vivo* injection of NR4A2 siRNA prevented the development of EAE by specifically inhibiting Th17 cell production of IL-17, but not affecting Th1 cells. Based on these findings, we propose that NR4A2 has direct effects on T cell pathogenicity or plays a critical role in continuous new Th17 differentiation and thus it orchestrates effector functions of Th17 cells in mediating autoimmune diseases.

Reagents that dampen the function of Th17 cells are of practical interest in clinical settings. Indeed, an IL-17A-specific antibody was efficacious in clinical trials of human psoriasis, uveitis, and rheumatoid arthritis [44]. Although the effects of IL-17 blockade on EAE appear to be only modest [45], [46], clinical trials are currently in progress to test IL-17A-specific antibody treatment in MS patients. As Th17 cells probably generate a range of proinflammatory cytokines, it is debatable how effective a therapy targeting a single cytokine may prove. NR4A2 appears to control various molecules regulated by IL-21 signalling and therefore a drug targeting NR4A2 may prove more effective than an antibody against a single cytokine.

We previously described that forced expression of NR4A2 in resting T cells led to a modest increase in IFN-γ production following nonspecific stimulation and suggested that NR4A2 might be involved in both Th1 and Th17 cell responses [27]. In contrast, we here reveal that only Th17 cells were found to express NR4A2 in the lesions of EAE, implying that NR4A2 plays a more important role in Th17 cells than in Th1 cells during autoimmune inflammation. However, it is not surprising to see a reduced IFN-γ production in NR4A2 knocked-down T cells *in vitro* (Fig. 2A), since IL-21 has potentials to modulate IFN-γ production under particular conditions [47]. An unexpected report was that the absence of NR4A2 led to increased expression of IFN-γ [37]. The authors argued that the increased production of IFN-γ was due to reduced activity of Foxp3^+^ regulatory T cells that are also dependent on NR4A2 [37]. However, this report does not contradict with our results, because in our *in vitro* experiments, we have explicitly removed regulatory T cells from the starting cell populations.

In agreement with previous studies [17], [48], our results showed that autocrine IL-21 production would promote IL-23R expression and subsequent production of IL-17 during Th17 cell differentiation. When NR4A2 upregulation was blocked by siRNA treatment, the production of IL-17 was greatly reduced, although RORγt expression was maintained, but exogenously added IL-21 restored IL-17 and IL-23R expression. Furthermore, NR4A2 also appeared to be required for the induction of the transcription factor that controls IL-21 secretion following Th17 differentiation, c-maf [41]. Moreover, lack of NR4A2 had little effect on RORγt expression, implying that this transcription factor is not associated with an IL-21-related pathway. It is interesting that IL-22 production is also maintained in the absence of NR4A2, despite the lack of IL-23R, suggesting that this cytokine could be produced via IL-23-independent pathways. TGF-β signals usually inhibit IL-22 production [16], however it is conceivable that these inhibitory signals are ineffective without NR4A2, thus allowing IL-22 secretion in the absence of increased IL-23R expression. Thus, NR4A2 modulation of Th17 cells may be limited to a role in the c-maf/IL-21 pathway, which ultimately controls IL-23R regulation and subsequent signalling. Given the critical role of IL-23 during pathogenic Th17 cell differentiation [16], the ability of NR4A2 siRNA to affect IL-23R expression by inhibiting IL-21 production cannot be ignored. It has been shown that IL-21 can drive Th17 differentiation [49] and thus enhance the initiation phase of EAE [50]. Therefore, the NR4A2-IL-21 pathway is particularly interesting as a therapeutic target. On the other hand, although a report claims that IL-21 is essential for Th17 differentiation [39], other reports showed Th17 differentiation in the absence of IL-21, albeit at a reduced level [14], and IL-17 production and EAE induction were not entirely blocked without IL-21 autocrine signals [51], [52]. The discrepancy regarding the role of IL-21 in Th17 cell induction remains to be fully understood and it is possible that absence of IL-21 signalling in gene knockout mice may be compensated by an alternative cytokine signalling pathway. However, our data may be explained by direct effects of NR4A2 on IL-23R upregulation as well as on the c-maf/IL-21 pathway.

In conclusion, our findings highlight the application of siRNA *in vivo* to modulate immunologic pathways that generate pathogenic autoimmune responses. Furthermore, our discovery of the key role of NR4A2 signalling in Th17 differentiation and our identification of the involvement of NR4A2 in generating autoimmune response *in vivo* suggest a new target for intervention in Th17-mediated autoimmune disease. This view is supported by our direct demonstration that manipulation of NR4A2 expression by siRNA treatment in established disease ameliorated clinical symptoms. Thus, future therapies targeting NR4A2 may prove highly effective in treating particular autoimmune diseases.

## Supporting Information

Figure S1 **EAE or EAU was induced in C57BL/6 mice by immunization with MOG_35–55_ or IRBP_1–20_ peptide in CFA.** EAE was scored clinically (A). CD4^+^ T cells were purified from the retina on the indicated days, and EAU disease severity was evaluated by flow cytometric enumeration of ocular infiltrates for EAU (B). Timepoints correspond to a minimum of 5 animals and data are representative of 3 independent experiments. A group of C57BL/6 mice received a low dose of STZ daily for 5 days. Clinical diabetes was tested by measurement of urine glucose level, with diabetes confirmed by consecutive urine glucose result of greater than ≥300 mg/dl (C). Plot C shows percentage diabetes. Other groups of C57BL/6 mice were immunized with peptides in CFA plus PTX either OVA_323–339_ (OVA/CFA) or MOG_35–55_ (MOG/CFA). On day 22, splenocytes and leukocytes isolated from the relevant target organ (ND, OVA/CFA; CNS, EAE; pancreas, STZ) were restimulated with 20 mg/ml of the immunizing peptide, or with soluble anti-CD3 (for STZ); after 96 hours, IL-17 and IFN-γ were measured in supernatants by ELISA (D). NR4A2 expression by blood T cells was also measured at a range of timepoints (E). Timepoints correspond to a minimum of 5 animals and data are representative of 2 independent experiments.
(TIF)

Figure S2 **Naïve CD4^+^ T cells were transfected by electroporation with NR4A2-specific siRNA or scrambled control siRNA.** Cells were then activated with 5 µg/ml plate-bound CD3-specific mAb and 0.5 µg/ml soluble CD28-specific mAb in the presence of 20 ng/ml IL-6, 20 ng/ml IL-23, and 3 ng/ml TGF-β. NR4A2 expression was assessed at a range of timepoints by RT PCR (A). Data are representative of 5 independent experiments. Cell proliferation of transfected cells following anti-CD3/anti-CD28 stimulation in the presence of Th1 (+10 ng/ml IL-12), Th17 (+20 ng/ml IL-6, 20 ng/ml IL-23, and 3 ng/ml TGF-β), or in the absence of polarizing cytokines was measured at 96 hours by the incorporation of ^3^H-thymidine. Data are representative of 2 independent experiments.
(TIF)

Figure S3 **Naïve CD4^+^ T cells transfected by electroporation with NR4A2-specific siRNA or scrambled control siRNA were activated with plate-bound CD3-specific mAb and soluble CD28-specific mAb in the presence of 10 µg/ml IFN-γ-specific and IL-4-specific mAb, with either 20 ng/ml IL-6, 2 ng/ml TGF-β (IL-6+ TGF-β) or with 10 ng/ml TGF-β (TGF-β).** Foxp3 expression at 96 hours as measured by real time PCR is shown in plot A. Data shown represent averages of 4 independent experiments. Naïve CD4^+^ T cells were transfected by electroporation with 2 siRNAs: either Foxp3-specific siRNA or relevant scrambled control siRNA and with either NR4A2-specific siRNA or relevant scrambled control siRNA. This yielded 4 cell types: 1) Foxp3 control/NR4A2 control (C/C); 2) Foxp3 control/NR4A2 siRNA (C/N); 3) Foxp3 siRNA/NR4A2 control (F/C); and 4) Foxp3 siRNA/NR4A2 siRNA (F/N). Cells were then activated with plate-bound CD3-specific mAb and soluble CD28-specific mAb in the presence of 10 µg/ml IFN-γ-specific and IL-4-specific mAb with either 20 ng/ml IL-6, 2 ng/ml TGF-β (IL-6+ TGF-β) or with 10 ng/ml TGF-β (TGF-β). Plot B shows IL-17 production from each of 4 siRNA-treated cell types at 96 hours as measured by ELISA. Data are representative of 2 independent experiments. siRNA, either NR4A2-specific or control, was stabilized in a collagen matrix and administered *i.v.* to groups of C57BL/6 mice at the time of EAE induction. At the indicated timepoints, CNS-infiltrating T cells were FACS-sorted and NR4A2 expression was assessed by RT PCR (A). CNS-infiltrating leukocytes from day 15 post-EAE induction from control or NR4A2 siRNA-treated mice were restimulated with PMA/ionomycin for 5 hours, and IL-17 and IFN-γ production were visualized by intracellular flow cytometric staining (B). Data are representative of 3 independent experiments
(TIF)

Figure S4 **siRNA, either NR4A2-specific or control, was stabilized in a collagen matrix and administered** ***i.v.*** **to groups of C57BL/6 mice at the time of EAE induction.** At the indicated timepoints, CNS-infiltrating T cells were FACS-sorted and NR4A2 expression was assessed by RT PCR (A). CNS-infiltrating leukocytes from day 15 post-EAE induction from control or NR4A2 siRNA-treated mice were restimulated with PMA/ionomycin for 5 hours, and IL-17 and IFN-γ production were visualized by intracellular flow cytometric staining (B). Data are representative of 3 independent experiments.
(TIF)

## References

[1] Sospedra M, Martin R (2005) Immunology of multiple sclerosis. Annu Rev Immunol 23: 683–747. 15771584 10.1146/annurev.immunol.23.021704.115707

[2] Bettelli E, Korn T, Kuchroo VK (2007) Th17: the third member of the effector T cell trilogy. Curr Opin Immunol 19: 652–657. 17766098 10.1016/j.coi.2007.07.020 PMC2288775

[3] Damsker JM, Hansen AM, Caspi RR (2010) Th1 and Th17 cells: adversaries and collaborators. Ann N Y Acad Sci 1183: 211–221. 20146717 10.1111/j.1749-6632.2009.05133.x PMC2914500

[4] Korn T, Oukka M, Kuchroo V, Bettelli E (2007) Th17 cells: effector T cells with inflammatory properties. Semin Immunol 19: 362–371. 18035554 10.1016/j.smim.2007.10.007 PMC2839934

[5] Goverman J (2009) Autoimmune T cell responses in the central nervous system. Nat Rev Immunol 9: 393–407. 19444307 10.1038/nri2550 PMC2813731

[6] Jager A, Dardalhon V, Sobel RA, Bettelli E, Kuchroo VK (2009) Th1, Th17, and Th9 effector cells induce experimental autoimmune encephalomyelitis with different pathological phenotypes. J Immunol 183: 7169–7177. 19890056 10.4049/jimmunol.0901906 PMC2921715

[7] Luger D, Silver PB, Tang J, Cua D, Chen Z, et al (2008) Either a Th17 or a Th1 effector response can drive autoimmunity: conditions of disease induction affect dominant effector category. J Exp Med 205: 799–810. 18391061 10.1084/jem.20071258 PMC2292220

[8] Steinman L (2008) A rush to judgment on Th17. J Exp Med 205: 1517–1522. 18591407 10.1084/jem.20072066 PMC2442627

[9] Man S, Ubogu EE, Ransohoff RM (2007) Inflammatory cell migration into the central nervous system: a few new twists on an old tale. Brain Pathol 17: 243–250. 17388955 10.1111/j.1750-3639.2007.00067.x PMC8095646

[10] Matusevicius D, Kivisakk P, He B, Kostulas N, Ozenci V, et al (1999) Interleukin-17 mRNA expression in blood and CSF mononuclear cells is augmented in multiple sclerosis. Mult Scler 5: 101–104. 10335518 10.1177/135245859900500206

[11] Hirota K, Duarte JH, Veldhoen M, Hornsby E, Li Y, et al (2011) Fate mapping of IL-17-producing T cells in inflammatory responses. Nat Immunol 12: 255–263. 21278737 10.1038/ni.1993 PMC3040235

[12] Bettelli E, Carrier Y, Gao W, Korn T, Strom TB, et al (2006) Reciprocal developmental pathways for the generation of pathogenic effector TH17 and regulatory T cells. Nature 441: 235–238. 16648838 10.1038/nature04753

[13] Korn T, Bettelli E, Oukka M, Kuchroo VK (2009) IL-17 and Th17 Cells. Annu Rev Immunol 27: 485–517. 19132915 10.1146/annurev.immunol.021908.132710

[14] Korn T, Bettelli E, Gao W, Awasthi A, Jager A, et al (2007) IL-21 initiates an alternative pathway to induce proinflammatory T(H)17 cells. Nature 448: 484–487. 17581588 10.1038/nature05970 PMC3805028

[15] Das J, Ren G, Zhang L, Roberts AI, Zhao X, et al (2009) Transforming growth factor beta is dispensable for the molecular orchestration of Th17 cell differentiation. J Exp Med 206: 2407–2416. 19808254 10.1084/jem.20082286 PMC2768861

[16] Ghoreschi K, Laurence A, Yang XP, Tato CM, McGeachy MJ, et al (2011) Generation of pathogenic T(H)17 cells in the absence of TGF-beta signalling. Nature 467: 967–971. 10.1038/nature09447 PMC3108066 20962846

[17] Cua DJ, Sherlock J, Chen Y, Murphy CA, Joyce B, et al (2003) Interleukin-23 rather than interleukin-12 is the critical cytokine for autoimmune inflammation of the brain. Nature 421: 744–748. 12610626 10.1038/nature01355

[18] McGeachy MJ, Cua DJ (2007) The link between IL-23 and Th17 cell-mediated immune pathologies. Semin Immunol 19: 372–376. 18319054 10.1016/j.smim.2007.10.012

[19] Law SW, Conneely OM, DeMayo FJ, O'Malley BW (1992) Identification of a new brain-specific transcription factor, NURR1. Mol Endocrinol 6: 2129–2135. 1491694 10.1210/mend.6.12.1491694

[20] Perlmann T, Wallen-Mackenzie A (2004) Nurr1, an orphan nuclear receptor with essential functions in developing dopamine cells. Cell Tissue Res 318: 45–52. 15340833 10.1007/s00441-004-0974-7

[21] Le WD, Xu P, Jankovic J, Jiang H, Appel SH, et al (2003) Mutations in NR4A2 associated with familial Parkinson disease. Nat Genet 33: 85–89. 12496759 10.1038/ng1066

[22] Pearen MA, Muscat GE (2010) Minireview: Nuclear Hormone Receptor 4A Signaling: Implications for Metabolic Disease. Mol Endocrinol 24: 1–13. 20392876 10.1210/me.2010-0015 PMC5417389

[23] Aherne CM, McMorrow J, Kane D, FitzGerald O, Mix KS, et al (2009) Identification of NR4A2 as a transcriptional activator of IL-8 expression in human inflammatory arthritis. Mol Immunol 46: 3345–3357. 19732956 10.1016/j.molimm.2009.07.019

[24] O'Kane M, Markham T, McEvoy AN, Fearon U, Veale DJ, et al (2008) Increased expression of the orphan nuclear receptor NURR1 in psoriasis and modulation following TNF-alpha inhibition. J Invest Dermatol 128: 300–310. 17671512 10.1038/sj.jid.5701023

[25] Saijo K, Winner B, Carson CT, Collier JG, Boyer L, et al (2009) A Nurr1/CoREST pathway in microglia and astrocytes protects dopaminergic neurons from inflammation-induced death. Cell 137: 47–59. 19345186 10.1016/j.cell.2009.01.038 PMC2754279

[26] Satoh J, Nakanishi M, Koike F, Miyake S, Yamamoto T, et al (2005) Microarray analysis identifies an aberrant expression of apoptosis and DNA damage-regulatory genes in multiple sclerosis. Neurobiol Dis 18: 537–550. 15755681 10.1016/j.nbd.2004.10.007

[27] Doi Y, Oki S, Ozawa T, Hohjoh H, Miyake S, et al (2008) Orphan nuclear receptor NR4A2 expressed in T cells from multiple sclerosis mediates production of inflammatory cytokines. Proc Natl Acad Sci U S A 105: 8381–8386. 18550828 10.1073/pnas.0803454105 PMC2426110

[28] Klemann C, Raveney BJ, Klemann AK, Ozawa T, von Horsten S, et al (2009) Synthetic retinoid AM80 inhibits Th17 cells and ameliorates experimental autoimmune encephalomyelitis. Am J Pathol 174: 2234–2245. 19389933 10.2353/ajpath.2009.081084 PMC2684188

[29] Raveney BJ, Copland DA, Nicholson LB, Dick AD (2008) Fingolimod (FTY720) as an acute rescue therapy for intraocular inflammatory disease. Arch Ophthalmol 126: 1390–1395. 18852417 10.1001/archopht.126.10.1390

[30] Copland DA, Wertheim MS, Armitage WJ, Nicholson LB, Raveney BJ, et al (2008) The clinical time-course of experimental autoimmune uveoretinitis using topical endoscopic fundal imaging with histologic and cellular infiltrate correlation. Invest Ophthalmol Vis Sci 49: 5458–5465. 18757507 10.1167/iovs.08-2348

[31] Mensah-Brown EP, Shahin A, Al-Shamisi M, Wei X, Lukic ML (2006) IL-23 leads to diabetes induction after subdiabetogenic treatment with multiple low doses of streptozotocin. Eur J Immunol 36: 216–223. 16358360 10.1002/eji.200535325

[32] Fraser JM, Janicki CN, Raveney BJ, Morgan DJ (2006) Abortive activation precedes functional deletion of CD8+ T cells following encounter with self-antigens expressed by resting B cells in vivo. Immunology 119: 126–133. 16796693 10.1111/j.1365-2567.2006.02414.x PMC1782339

[33] Raveney BJ, Copland DA, Dick AD, Nicholson LB (2009) TNFR1-dependent regulation of myeloid cell function in experimental autoimmune uveoretinitis. J Immunol 183: 2321–2329. 19635911 10.4049/jimmunol.0901340

[34] Shao H, Liao T, Ke Y, Shi H, Kaplan HJ, et al (2006) Severe chronic experimental autoimmune uveitis (EAU) of the C57BL/6 mouse induced by adoptive transfer of IRBP1–20-specific T cells. Exp Eye Res 82: 323–331. 16125173 10.1016/j.exer.2005.07.008

[35] Elias D, Prigozin H, Polak N, Rapoport M, Lohse AW, et al (1994) Autoimmune diabetes induced by the beta-cell toxin STZ. Immunity to the 60-kDa heat shock protein and to insulin. Diabetes 43: 992–998. 8039607 10.2337/diab.43.8.992

[36] Zhou L, Lopes JE, Chong MM, Ivanov, II, Min R, et al (2008) TGF-beta-induced Foxp3 inhibits T(H)17 cell differentiation by antagonizing RORgammat function. Nature 453: 236–240. 18368049 10.1038/nature06878 PMC2597437

[37] Sekiya T, Kashiwagi I, Inoue N, Morita R, Hori S, et al (2011) The nuclear orphan receptor Nr4a2 induces Foxp3 and regulates differentiation of CD4+ T cells. Nat Commun 2: 269. 21468021 10.1038/ncomms1272 PMC3104557

[38] Zhou L, Ivanov, II, Spolski R, Min R, Shenderov K, et al (2007) IL-6 programs T(H)-17 cell differentiation by promoting sequential engagement of the IL-21 and IL-23 pathways. Nat Immunol 8: 967–974. 17581537 10.1038/ni1488

[39] Nurieva R, Yang XO, Martinez G, Zhang Y, Panopoulos AD, et al (2007) Essential autocrine regulation by IL-21 in the generation of inflammatory T cells. Nature 448: 480–483. 17581589 10.1038/nature05969

[40] Ivanov, II, Zhou L, Littman DR (2007) Transcriptional regulation of Th17 cell differentiation. Semin Immunol 19: 409–417. 18053739 10.1016/j.smim.2007.10.011 PMC2696342

[41] Bauquet AT, Jin H, Paterson AM, Mitsdoerffer M, Ho IC, et al (2009) The costimulatory molecule ICOS regulates the expression of c-Maf and IL-21 in the development of follicular T helper cells and TH-17 cells. Nat Immunol 10: 167–175. 19098919 10.1038/ni.1690 PMC2742982

[42] Veldhoen M, Hirota K, Westendorf AM, Buer J, Dumoutier L, et al (2008) The aryl hydrocarbon receptor links TH17-cell-mediated autoimmunity to environmental toxins. Nature 453: 106–109. 18362914 10.1038/nature06881

[43] Takeshita F, Minakuchi Y, Nagahara S, Honma K, Sasaki H, et al (2005) Efficient delivery of small interfering RNA to bone-metastatic tumors by using atelocollagen in vivo. Proc Natl Acad Sci U S A 102: 12177–12182. 16091473 10.1073/pnas.0501753102 PMC1183487

[44] Hueber W, Patel DD, Dryja T, Wright AM, Koroleva I, et al (2011) Effects of AIN457, a fully human antibody to interleukin-17A, on psoriasis, rheumatoid arthritis, and uveitis. Sci Transl Med 2: 52ra72. 10.1126/scitranslmed.3001107 20926833

[45] Hofstetter HH, Ibrahim SM, Koczan D, Kruse N, Weishaupt A, et al (2005) Therapeutic efficacy of IL-17 neutralization in murine experimental autoimmune encephalomyelitis. Cell Immunol 237: 123–130. 16386239 10.1016/j.cellimm.2005.11.002

[46] Komiyama Y, Nakae S, Matsuki T, Nambu A, Ishigame H, et al (2006) IL-17 plays an important role in the development of experimental autoimmune encephalomyelitis. J Immunol 177: 566–573. 16785554 10.4049/jimmunol.177.1.566

[47] Monteleone G, Monteleone I, Fina D, Vavassori P, Del Vecchio Blanco G, et al (2005) Interleukin-21 enhances T-helper cell type I signaling and interferon-gamma production in Crohn's disease. Gastroenterology 128: 687–694. 15765404 10.1053/j.gastro.2004.12.042

[48] Langrish CL, Chen Y, Blumenschein WM, Mattson J, Basham B, et al (2005) IL-23 drives a pathogenic T cell population that induces autoimmune inflammation. J Exp Med 201: 233–240. 15657292 10.1084/jem.20041257 PMC2212798

[49] Leonard WJ, Spolski R (2005) Interleukin-21: a modulator of lymphoid proliferation, apoptosis and differentiation. Nat Rev Immunol 5: 688–698. 16138102 10.1038/nri1688

[50] Vollmer TL, Liu R, Price M, Rhodes S, La Cava A, et al (2005) Differential effects of IL-21 during initiation and progression of autoimmunity against neuroantigen. J Immunol 174: 2696–2701. 15728477 10.4049/jimmunol.174.5.2696

[51] Coquet JM, Chakravarti S, Smyth MJ, Godfrey DI (2008) Cutting edge: IL-21 is not essential for Th17 differentiation or experimental autoimmune encephalomyelitis. J Immunol 180: 7097–7101. 18490706 10.4049/jimmunol.180.11.7097

[52] Sonderegger I, Kisielow J, Meier R, King C, Kopf M (2008) IL-21 and IL-21R are not required for development of Th17 cells and autoimmunity in vivo. Eur J Immunol 38: 1833–1838. 18546146 10.1002/eji.200838511

