# Supplementary figures and images for: Nuclear Receptor NR4A2 Orchestrates Th17 Cell-Mediated Autoimmune Inflammation via IL-21 Signalling
Source: PLoS One. 2013 Feb 21;8(2):e56595. doi: 10.1371/journal.pone.0056595 (PMC3578929; doi:10.1371/journal.pone.0056595)

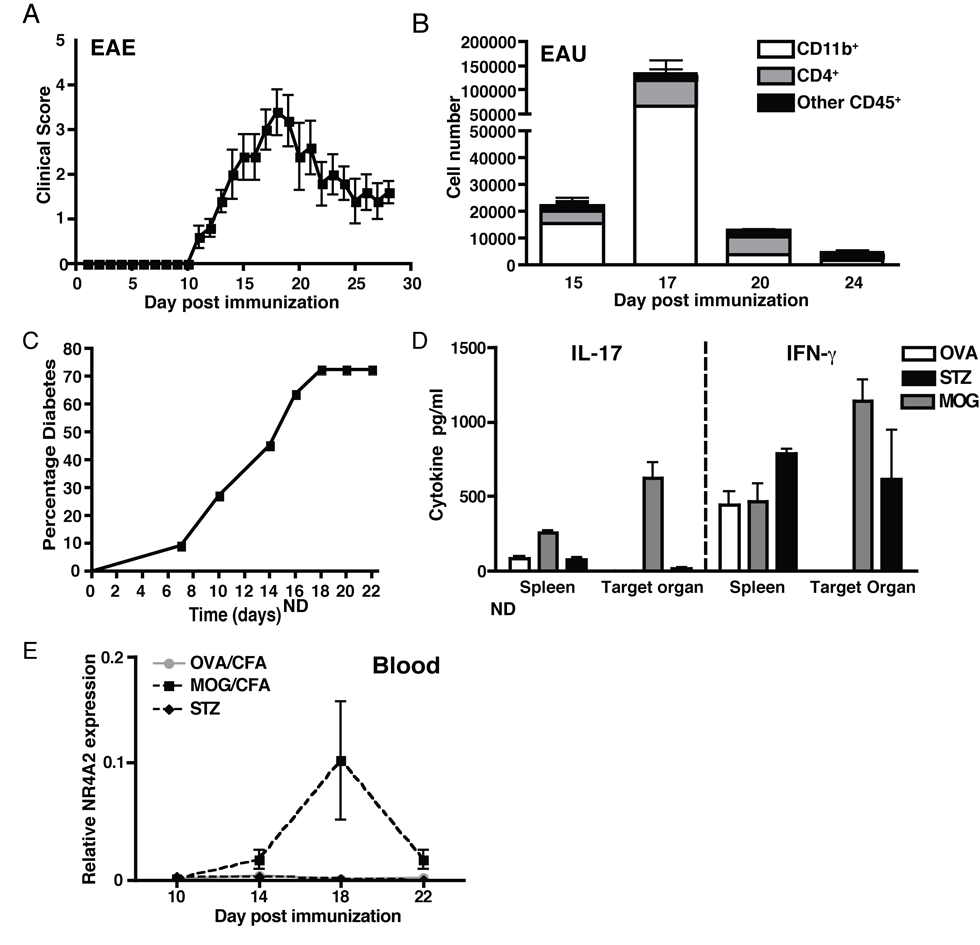

Supplement: Figure S1 — EAE or EAU was induced in C57BL/6 mice by immunization with MOG35–55 or IRBP1–20 peptide in CFA. EAE was scored clinically (A). CD4+ T cells were purified from the retina on the indicated days, and EAU disease severity was evaluated by flow cytometric enumeration of ocular infiltrates for EAU (B). Timepoints correspond to a minimum of 5 animals and data are representative of 3 independent experiments. A group of C57BL/6 mice received a low dose of STZ daily for 5 days. Clinical diabetes was tested by measurement of urine glucose level, with diabetes confirmed by consecutive urine glucose result of greater than ≥300 mg/dl (C). Plot C shows percentage diabetes. Other groups of C57BL/6 mice were immunized with peptides in CFA plus PTX either OVA323–339 (OVA/CFA) or MOG35–55 (MOG/CFA). On day 22, splenocytes and leukocytes isolated from the relevant target organ (ND, OVA/CFA; CNS, EAE; pancreas, STZ) were restimulated with 20 mg/ml of the immunizing peptide, or with soluble anti-CD3 (for STZ); after 96 hours, IL-17 and IFN-γ were measured in supernatants by ELISA (D). NR4A2 expression by blood T cells was also measured at a range of timepoints (E). Timepoints correspond to a minimum of 5 animals and data are representative of 2 independent experiments. (TIF) [file pone.0056595.s001.tif]

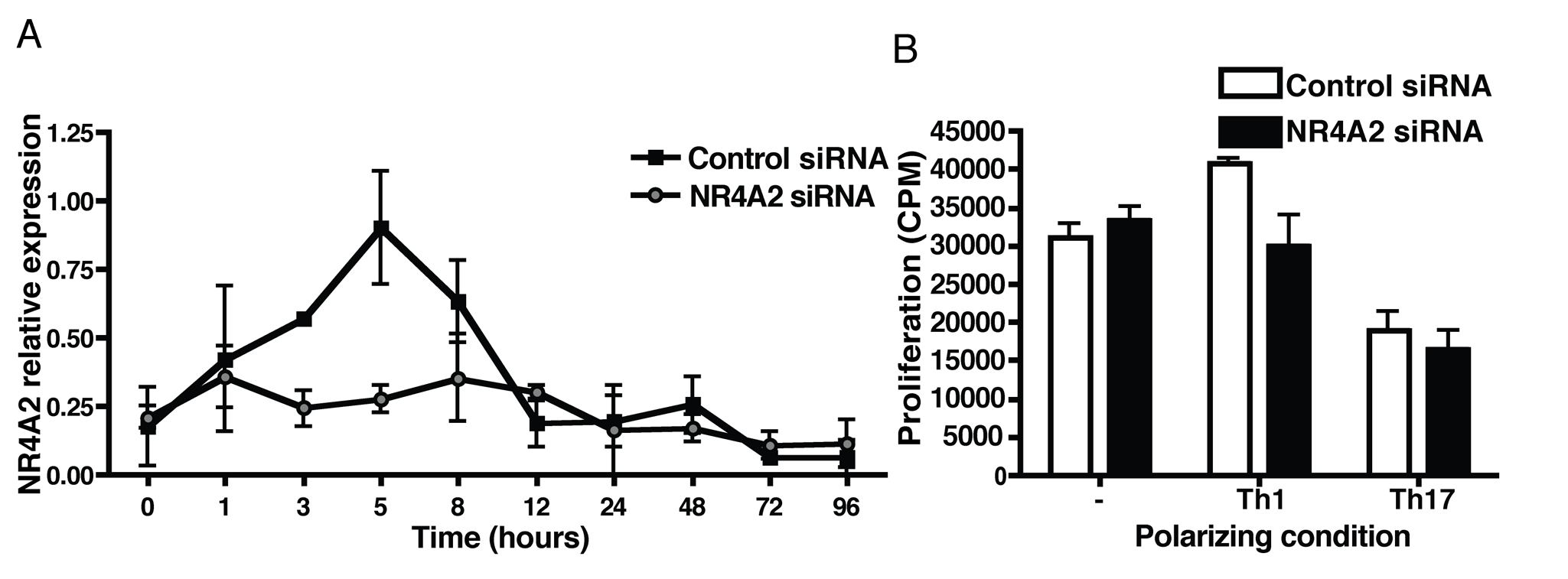

Supplement: Figure S2 — Naïve CD4+ T cells were transfected by electroporation with NR4A2-specific siRNA or scrambled control siRNA. Cells were then activated with 5 µg/ml plate-bound CD3-specific mAb and 0.5 µg/ml soluble CD28-specific mAb in the presence of 20 ng/ml IL-6, 20 ng/ml IL-23, and 3 ng/ml TGF-β. NR4A2 expression was assessed at a range of timepoints by RT PCR (A). Data are representative of 5 independent experiments. Cell proliferation of transfected cells following anti-CD3/anti-CD28 stimulation in the presence of Th1 (+10 ng/ml IL-12), Th17 (+20 ng/ml IL-6, 20 ng/ml IL-23, and 3 ng/ml TGF-β), or in the absence of polarizing cytokines was measured at 96 hours by the incorporation of 3H-thymidine. Data are representative of 2 independent experiments. (TIF) [file pone.0056595.s002.tif]

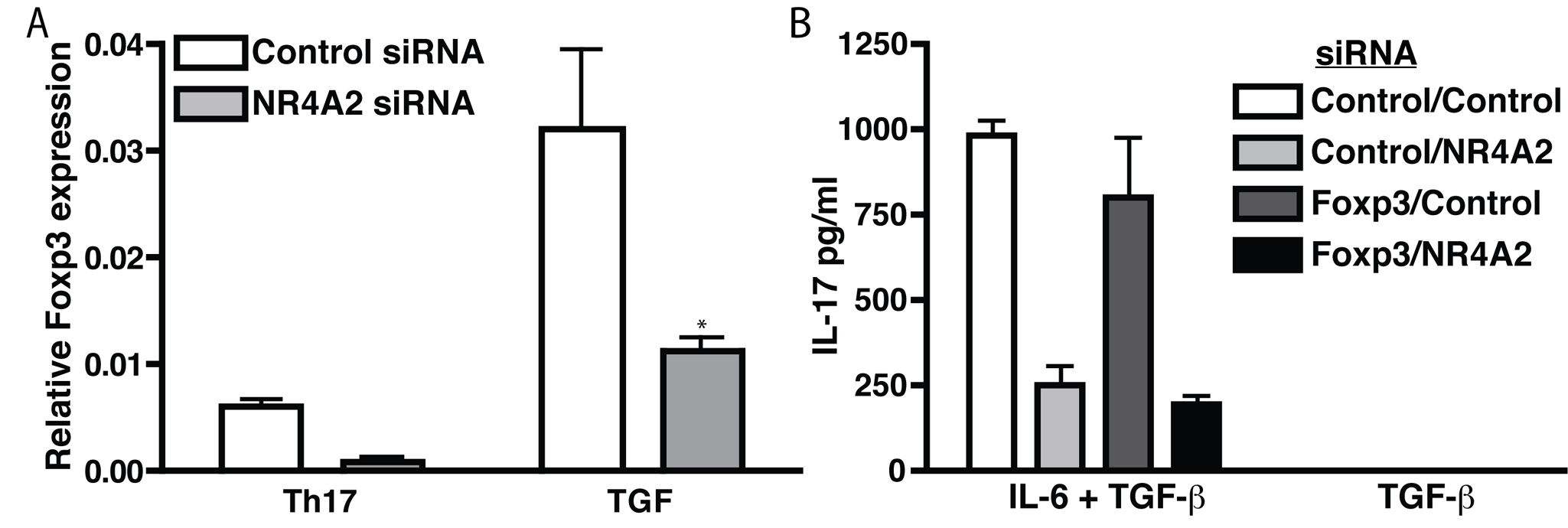

Supplement: Figure S3 — Naïve CD4+ T cells transfected by electroporation with NR4A2-specific siRNA or scrambled control siRNA were activated with plate-bound CD3-specific mAb and soluble CD28-specific mAb in the presence of 10 µg/ml IFN-γ-specific and IL-4-specific mAb, with either 20 ng/ml IL-6, 2 ng/ml TGF-β (IL-6+ TGF-β) or with 10 ng/ml TGF-β (TGF-β). Foxp3 expression at 96 hours as measured by real time PCR is shown in plot A. Data shown represent averages of 4 independent experiments. Naïve CD4+ T cells were transfected by electroporation with 2 siRNAs: either Foxp3-specific siRNA or relevant scrambled control siRNA and with either NR4A2-specific siRNA or relevant scrambled control siRNA. This yielded 4 cell types: 1) Foxp3 control/NR4A2 control (C/C); 2) Foxp3 control/NR4A2 siRNA (C/N); 3) Foxp3 siRNA/NR4A2 control (F/C); and 4) Foxp3 siRNA/NR4A2 siRNA (F/N). Cells were then activated with plate-bound CD3-specific mAb and soluble CD28-specific mAb in the presence of 10 µg/ml IFN-γ-specific and IL-4-specific mAb with either 20 ng/ml IL-6, 2 ng/ml TGF-β (IL-6+ TGF-β) or with 10 ng/ml TGF-β (TGF-β). Plot B shows IL-17 production from each of 4 siRNA-treated cell types at 96 hours as measured by ELISA. Data are representative of 2 independent experiments. siRNA, either NR4A2-specific or control, was stabilized in a collagen matrix and administered i.v. to groups of C57BL/6 mice at the time of EAE induction. At the indicated timepoints, CNS-infiltrating T cells were FACS-sorted and NR4A2 expression was assessed by RT PCR (A). CNS-infiltrating leukocytes from day 15 post-EAE induction from control or NR4A2 siRNA-treated mice were restimulated with PMA/ionomycin for 5 hours, and IL-17 and IFN-γ production were visualized by intracellular flow cytometric staining (B). Data are representative of 3 independent experiments (TIF) [file pone.0056595.s003.tif]

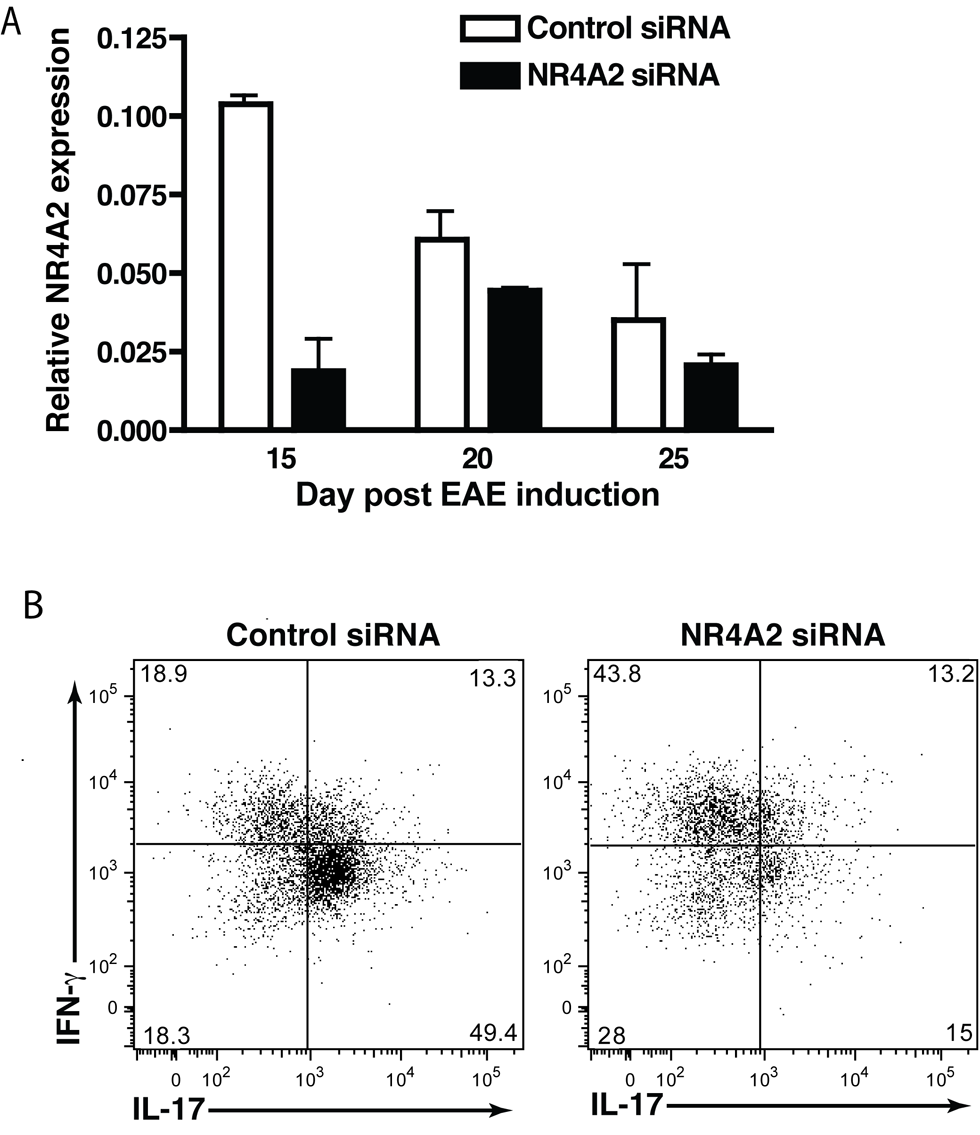

Supplement: Figure S4 — siRNA, either NR4A2-specific or control, was stabilized in a collagen matrix and administered i.v. to groups of C57BL/6 mice at the time of EAE induction. At the indicated timepoints, CNS-infiltrating T cells were FACS-sorted and NR4A2 expression was assessed by RT PCR (A). CNS-infiltrating leukocytes from day 15 post-EAE induction from control or NR4A2 siRNA-treated mice were restimulated with PMA/ionomycin for 5 hours, and IL-17 and IFN-γ production were visualized by intracellular flow cytometric staining (B). Data are representative of 3 independent experiments. (TIF) [file pone.0056595.s004.tif]
